# Traumatic Effects on Epigenetic Aging Extends Across Generations: A Study of Holocaust Survivors and Their Descendants

**DOI:** 10.1192/j.eurpsy.2026.10246

**Published:** 2026-07-31

**Authors:** A. Shrira, D. Cheishvili, M. Scharf, Y. Palgi, L. Ayalon, M. Bensimon, T. Rosenbloom, E. Bodner, M. Szyf, G. Yadid

**Affiliations:** 1 Department of Social and Health Sciences, Bar-Ilan University, Ramat Gan, Israel; 2 EpiMedTech Global, Singapore, Singapore; 3 HKG Epitherapeutics Inc, Hong Kong, China; 4 McGill University, Montreal, Canada; 5 University of Haifa, Haifa; 6 Bar-Ilan University, Ramat Gan, Israel

## Abstract

**Introduction:**

Experiencing severe trauma can have lasting effects that continue into old age, often leading to physical health issues. Severe trauma can also affect future generations within a family. Such intergenerational effects may result from biological dysregulation caused by epigenetic processes, including modifications of DNA methylation. However, it remains uncertain whether the effects of parental trauma are passed down through generations to influence the biological aging of descendants.

**Objectives:**

In our studies, we focused on epigenetic age, a biological measure of aging based on changes in DNA methylation that happen over time. Epigenetic age can differ from chronological age and is thought to reflect the body’s physiological condition. We aimed to examine whether Holocaust exposure and its psychological effects are linked to epigenetic age across two generations: those who lived during WWII (G1) and their children born after the war (G2).

**Methods:**

In Study 1, we examined a cohort of 20 G2 to Holocaust survivors (mean age=65). We explored whether their symptoms and their perceptions of their parents’ PTSD symptoms relate to epigenetic accelerated aging (EAA), calculated as the difference between epigenetic age and chronological age. In Study 2, we analyzed a larger cohort consisting of 50 parent-offspring dyads: G1 and G2 (mean age=87 and 56, respectively), some from Holocaust survivor families and others from non-Holocaust Jewish families. We investigated whether Holocaust G1 and G2 differ in EAA compared to their non-Holocaust counterparts. Additionally, we examined whether PTSD symptoms, other symptoms, and parental behaviors are associated with EAA across generations.

**Results:**

Study 1 found that Holocaust G2 PTSD symptoms and perceived maternal PTSD were linked to higher EAA. Study 2 confirmed some of these findings. Moreover, although there was no significant difference in EAA between the G1 groups, a high level of traumatic exposure during WWII was associated with increased EAA. Additionally, Holocaust G2 individuals exhibited higher EAA compared to non-Holocaust G2. Negative parental behaviors (reported by G1) and childhood abuse and neglect (reported by G2) were connected to higher EAA in G2.

**Conclusions:**

The current findings suggest that characteristics of Holocaust G1 significantly influence the epigenetic aging of their offspring. Connections to Holocaust exposure, parental behavior, and childhood abuse suggest traumatic aftereffects in G1 may leave a lasting imprint on their descendants’ biological aging processes. Future research on epigenetic aging may improve preventive strategies addressing intergenerational effects of trauma and help evaluate the effectiveness of interventions aimed at managing age-related health issues.

**Disclosure of Interest:**

None Declared

